# A multifunctional ‘golden cicada’ nanoplatform breaks the thermoresistance barrier to launch cascade augmented synergistic effects of photothermal/gene therapy

**DOI:** 10.1186/s12951-023-01983-3

**Published:** 2023-07-17

**Authors:** Wen Yang, Ning Wang, Jin Yang, Chao Liu, Shuang Ma, Xiye Wang, Wenzhen Li, Meiling Shen, Qinjie Wu, Changyang Gong

**Affiliations:** Department of Biotherapy, Cancer Center and State Key Laboratory of Biotherapy, West China Hospital, Sichuan University, Chengdu, 610041 China

**Keywords:** Gene delivery system, Photothermal therapy, Thermoresistance, Heat shock protein 70, Cascade augmented synergistic effects

## Abstract

**Background:**

Photothermal therapy (PTT) is taken as a promising strategy for cancer therapy, however, its applicability is hampered by cellular thermoresistance of heat shock response and insufficient accumulation of photothermal transduction agents in the tumor region. In consideration of those limitations, a multifunctional “Golden Cicada” nanoplatform (MGCN) with efficient gene delivery ability and excellent photothermal effects is constructed, overcoming the thermoresistance of tumor cells and improving the accumulation of indocyanine green (ICG).

**Results:**

Down-regulation of heat shock protein 70 (HSP70) makes tumor cells more susceptible to PTT, and a better therapeutic effect is achieved through such cascade augmented synergistic effects. MGCN has attractive features with prolonged circulation in blood, dual-targeting capability of CD44 and sialic acid (SA) receptors, and agile responsiveness of enzyme achieving size and charge double-variable transformation. It proves that, on the one hand, MGCN performs excellent capability for HSP70-shRNA delivery, resulting in breaking the cellular thermoresistance mechanism, on the other hand, ICG enriches in tumor site specifically and possesses a great thermal property to promoted PTT.

**Conclusions:**

In short, MGCN breaks the protective mechanism of cellular heat stress response by downregulating the expression of HSP70 proteins and significantly augments synergistic effects of photothermal/gene therapy *via* cascade augmented synergistic effects.

**Supplementary Information:**

The online version contains supplementary material available at 10.1186/s12951-023-01983-3.

## Background

Photothermal therapy (PTT), a promising method for cancer treatment, is recognized to convert near-infrared (NIR) light energy to heat by using photothermal transduction agents (PTAs) and inhibit tumor cells rapidly with increasing temperature surrounding tumor [1–3]. It shows many advantages such as low cost, minimal side effects and noninvasive therapeutic modality for tumor [4, 5]. However, its applicability is constrained by some drawbacks [6, 7]. For example, heat shock protein 70 (HSP70) will upregulate to endow cells with thermoresistance and protect them from hyperthermia damage when cells are stimulated with toxic chemicals, heavy metals and especially heat [8–10]. This self-protection and anti-apoptotic ability result in a significant reduction of therapeutic effect of PTT. Along with the development of gene therapy, RNA interference-mediated short hairpin RNA (shRNA) against HSP70 is a powerful technology by the way of specific silencing HSP70 expression, thereby breaking the protective mechanism of heat shock response and making tumor cells more susceptible to PTT [11, 12]. In this case, gene therapy is in a position to overcome cellular thermoresistance and compensate for the weakness of PTT.

PTT capitalizes PTAs’ properties to convert optical energy into thermal energy, raising the local temperature to inhibit tumor cells rapidly. However, one shortcoming of PTAs is the lack of precise tumor targeting. Indocyanine green (ICG), a widely used cyanine photothermal conversion reagent, has obtained Food and Drug Administration (FDA) affirmation of clinical diagnostic for its low cytotoxicity and great biocompatibility in vivo, but insufficient tissue-selectivity limits its applications in PTT [6, 13, 14]. A viable strategy is to modify ICG on a tumor-targeting delivery vector, which could be masterfully designed to overcome biological barriers and accumulate in tumor site through enhanced permeability, retention effect and receptor-ligand mediated specific targeting ability. In this way, it increases the sufficient accumulation of ICG in tumor site, thereby enhancing the photothermal effect [15–17].

However, delivery vectors are required to overcome biological hurdles such as blood interference, non-specific phagocytosis and endo/lysosomal degradation, then achieve efficient HSP70-shRNA delivery into tumors [18–21]. It is a complex multi-step process, so a capable delivery vector is urgently needed. Polyethylenimine (PEI) are widely used as non-viral gene delivery vectors due to its unique features including high ionic charge density to condense with DNA, effectively internalized by tumor cells, and the proton sponge effect which could achieve DNA endosomal [19, 22, 23]. However, the transfection efficacy and cytotoxicity of PEI are strongly associated with the molecular weight. Low molecular weight PEI has lower toxic, but its transfection efficacy is also decrease with molecular weight [24]. Chemical modifications are taken as a powerful strategy to increase transfection efficacy of low molecular weight PEI [25–28]. 3-fluoro-4-carboxyphenylboronic acid (FPBA) is a low cytotoxicity molecule which is capable for generating reversible covalent cyclic boronic esters with diols such as sialic acid (SA) overexpressed on the cell [29, 30]. Fluorination existing in intergenic sites possesses electron-withdrawing properties and leads the ionization constants (pKa) decrease, thus making boronic acid more suitable for interacting with diols under physiological conditions (pH 7.4) [31–36]. Hence, we hypothesize that FPBA modified with low molecular weight PEI would achieve better transfection efficiency.

**Scheme 1:**
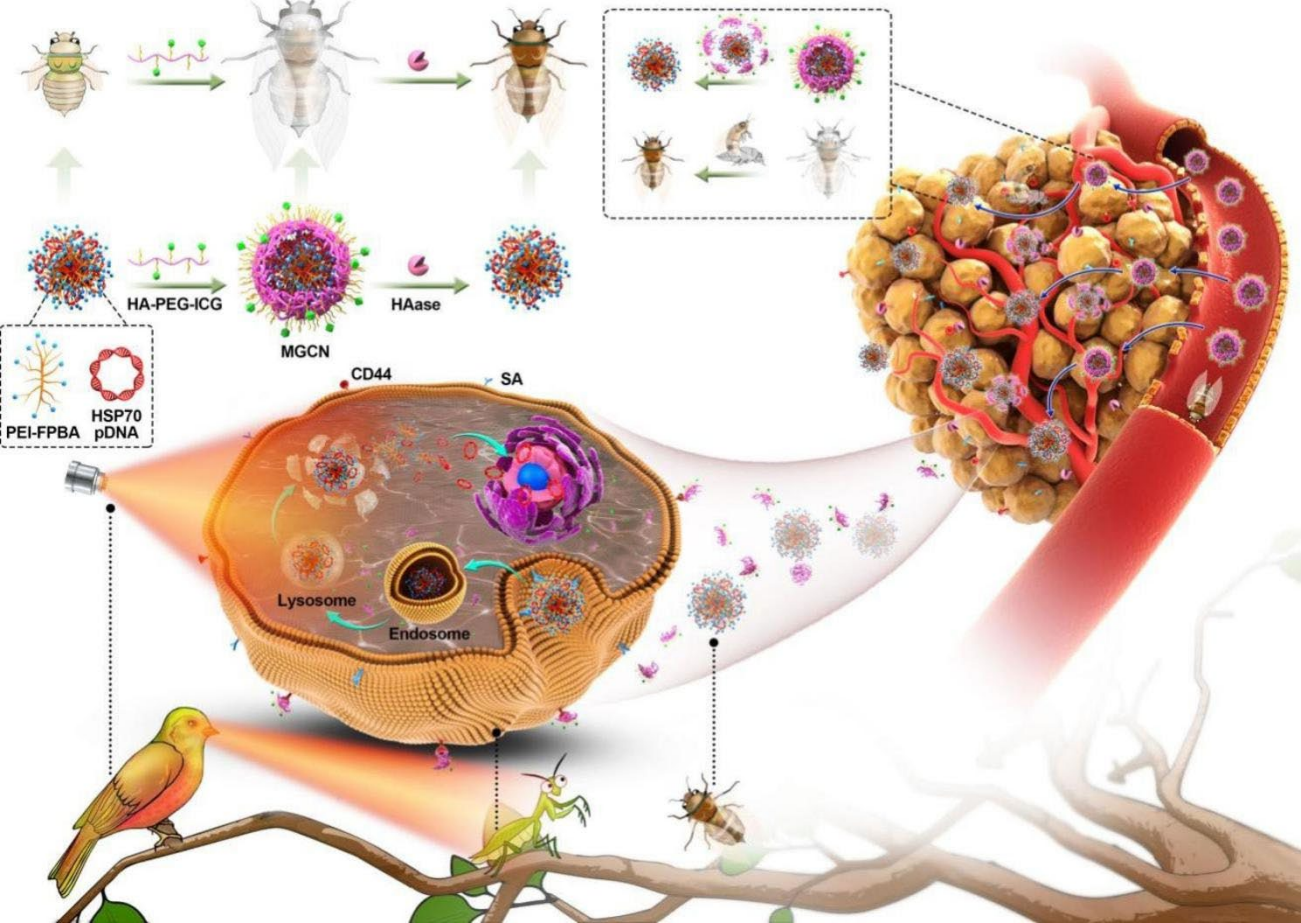
Schematic illustration of multifunctional “Golden Cicada” nanoplatform for cancer synergistic photothermal/gene therapy. The core-shell structured MGCN was constructed in a two-step electrostatic interacted way. The MGCN overcame physiological barriers to reach the tumor site specifically, it possessed properties of efficient gene delivery and excellent photothermal effects

Here, we presented a core-shell structure-based gene delivery system possessing properties of efficient gene delivery and excellent photothermal effects. The core was a cationic nanocomplex made from HSP70-shRNA plasmids and low molecular weight PEI modified with FPBA (PEI-FPBA), and then the core was coated with a shell which was consisted of hyaluronic acid (HA) and further modified with polyethylene glycol (PEG) and indocyanine green (ICG). When these nanocomplexes circulated in vivo, the modified shell HA-PEG-ICG (HPI) made them stealthy in circulation resulting in increased prolongation time, and achieved targeting for CD44 receptors which overexpressed on the cell surface [37–40]. With the assistance of targeting ability of nanocomplexes, ICG could enhance its efficient accumulation in the tumor. Subsequently, the shell detached from the core owing to its hyaluronidase response property, and further promoted intracellular accumulation of ICG. Detachment of the shell made nanocomplexes achieve size and charge double-variable transformation, and target for SA receptors for better cellular [20, 41, 42]. Nanocomplexes overcame physiological barriers to reach the tumor site specifically and achieved efficient functional gene delivery. HSP70-shRNA downregulated HSP70 protein resulting in breaking thermoresistance of cells. After that, laser irradiation was given, tumor cells were effectively inhibited through ICG-mediated photothermal effects. We vividly summarized this treatment process with an ancient proverb “The mantis stalks the cicada, unaware of the canaries behind”, nanocomplexes (Golden Cicadas) were “eaten” by tumors (Mantis) and then tumors were “killed” by photothermal therapy (Canaries) (Scheme 1). This cascade process with “Golden Cicadas” nanoplatform acted as a powerful strategy for overcoming thermoresistance and increasing the accumulation of ICG in tumor site, finally, enhancing the treatment efficacy of PTT.

## Methods

### Materials

Branched polyethyleneimine 1.8 kDa (PEI 1.8K) and branched polyethyleneimine 25 kDa (PEI 25K) were provided by Alfa Aesar (U.S.A.). Sodium hyaluronic acid (HA) 3.5 kDa was purchased from Shandong Freda Biochem Co. Ltd. (Shandong, China). BOC-NH-PEG-NH_2_ was purchased from Pensure Biotechnology Co. Ltd. (Shanghai, China). 3-fluoro-4-carboxyphyenylboronic acid (FPBA), N-hydroxysuccinimide (NHS), N, N’-dicyclohexylcarbodiimide (DCC), 1-(3-dimethylaminopropyl)-3-ethylcarbodiimide hydrochloride (EDCI), dimethyl sulfoxide (DMSO) and other chemicals were obtained from Aladdin (Shanghai, China). Indocyanine green was provided by Ruixi Biotechnology Co. Ltd. (Xi’an, China). Lipofetamine 3000 and YOYO-1 were purchased from Invitrogen (U.S.A.). LysoTracker Red and DAPI were obtained from Beyotime Biotechnology (Shanghai, China). Plasmids were designed and constructed by Fubio Biotechnology Co. Ltd. (Jiangsu, China). Annexin V-FITC/ Propidium (PI) apoptosis kit was bought from Beijing4A Biotechnology Co. Ltd. (Beijing, China). LIVE/DEAD viability/cytotoxicity kit and TUNEL apoptosis kit were provided by Vazyme Biotechnology Co. Ltd. (Nanjing, China). HSP70 antibody was acquired from Boster Biological Technology Co. Ltd. (Wuhan, China). Antibody β-actin was bought from Santa Cruz Biotechnology Co. Ltd. (U.S.A.)

### Cell culture and animals

B16-F10 mouse melanoma cells, L929 mouse fibroblasts cells and A375 human malignant melanoma cells were provided by American Type and Culture Collection (ATCC, U.S.A.). Dulbecco’s modified Eagle’s medium (Gibco, U.S.A.) containing 10% fetal bovine serum (Gibco, U.S.A.), 1% streptomycin and 1% penicillin was used for cell culture. Cells were maintained at 37 °C, 5% CO_2_ atmosphere.

C57BL/6 mice (female, aged 6–8 weeks) and BALB/c-nu mice (female, aged 6–8 weeks) were provided by Beijing HFK Bioscience Co. Ltd. (Beijing, China). All mice used under the study were housed individually in ventilated cages and maintained in a temperature-controlled environment (22 °C), food and water were accessible ad libitum. All animal experiments were approved by the Institutional Animal Care and Treatment Committee of Sichuan University (Chengdu, China).

### Synthesis and characterization of PEI-FPBA

PEI-FPBA was produced by grafting different mass ratios of FPBA onto PEI 1.8 K. Briefly, 40 mg, 101 mg, 141 mg and 202 mg of FPBA were dissolved respectively in DMSO and then activated by DCC (molar ratio was 1:1.5) and NHS (molar ratio was 1:1.5), solution was stirred at 25℃ for up to 8 h. 200 mg of PEI 1.8 K dissolved in DMSO was slowly added to the activated FPBA solution and stirred at 25℃ for 72 h. The yielding materials were dialyzed for 72 h using a 1 kDa MWCO dialysis bag against distilled water and lyophilized to obtain PEI-FPBA. The obtained products were characterized by ^*1*^* H* nuclear magnetic resonance (^*1*^* H* NMR) spectra and further analyzed by Fourier transform infrared (FT-IR) spectra.

### Synthesis and characterization of HA-PEG-ICG

HA-PEG-ICG (HPI) was achieved by conjugating polyethylene glycol 2000 (PEG_2000_) and indocyanine green (ICG) onto HA. Succinctly, 5.65 mg of EDCI, 3.4 mg of NHS and 11.4 mg of ICG were solubilized in DCM. The solution was stirred at 25 °C for 4 h to activate carboxyl groups of ICG. 35.3 mg of BOC-NH-PEG-NH_2_ was dissolved in DCM was added, and stirring was continued for 48 h at 25 °C. Then the BOC protecting group was removed through TFA/DCM at 25 °C for 4 h. Subsequently, the yielding materials were dialyzed for 48 h using a 3.5 kDa MWCO dialysis bag against distilled water and lyophilized to obtain the intermediate product NH_2_-PEG-ICG.

8.6 mg of EDCI, 5.59 mg of NHS and 10 mg of HA were added to 2-(N-morpholino) ethane sulfonic acid (MES) buffer, the reaction solution was stirred for 4 h. Subsequently, 28.8 mg of the intermediate product NH_2_-PEG-ICG was added, and the solution was continued stirring for 72 h. The yielding materials were dialyzed for 72 h using a 3.5 kDa MWCO dialysis bag against distilled water and lyophilized to obtain the final product HA-PEG-ICG. Characterizations of HPI, HA, BOC-NH-PEG-NH_2_ and ICG were identified by ^*1*^* H* NMR spectroscopy and FT-IR spectroscopy.

### Preparation and characterization of MGCN

PEI-FPBA/HSP70-shRNA nanocomplexes were prepared by mixing plasmids with PEI-FPBA solution at a mass ratio of 1:10 and incubating for 30 min. Then HPI solution was added to form HPI/PEI-FPBA/HSP70-shRNA nanocomplexes (HPI:PEI-FPBA:HSP70-shRNA = 40:10:1). The hydrodynamic size and zeta potential of PEI-FPBA/HSP70-shRNA and HPI/PEI-FPBA/HSP70-shRNA were analyzed with Malvern Zetasizer (Malvern, U.K.). Morphologies were assessed by transmission electron microscopy (TEM) (JEOL, Japan). For investigating the enzymatic sensitivity of MGCN, hyaluronidase simulating the tumor microenvironment (TME) was added to the solution. Then the hydrodynamic size, zeta potential and morphologies of MGCN were measured as described previously.

To determine the condensation ability of PEI-FPBA with HSP70-shRNA, agarose gel retardation assay was performed. PEI-FPBA and plasmids were mixed at different mass ratios of 0.5:1, 1:1, 2:1, 5:1, 10:1, 15:1 and 20:1. The nanocomplexes were prepared by mixing plasmids with PEI-FPBA solution as mentioned before. Nanocomplexes were added to agarose gel (1%) and separated at 120 V for 30 min in TAE buffer. The agarose gel with Gel Red staining was finally visualized.

In order to evaluate stability of MGCN in aqueous solution, free ICG and MGCN (containing equivalent amount of ICG) were stored for 72 h at 25℃ in the dark. At various time intervals (0 h, 24 h, 48 and 72 h), ultraviolet–visible (UV–vis) spectrophotometer (Shimadzu, Japan) was utilized to measure the samples absorption at 780 nm.

### In vitro cytotoxicity analysis

The cytotoxicities of PEI-FPBA and HPI were evaluated in B16-F10 cells and L929 cells through MTT experiment. PEI 1.8 K and PEI 25 K were assessed as controls. Briefly, cells were plated on 96-well plates and pre-maintained at 37 °C. Then they were cultured with PEI-FPBA, HPI, PEI 1.8 K and PEI 25 K at various concentrations (0 µg/mL – 40 µg/mL) for additional 24 h, respectively. After different treatments, the media was discarded, MTT was added and maintained with cells. Absorbance value was determined at 570 nm.

### Cellular uptake assay

HSP70-shRNA was labeled with YOYO-1 through specific covalent binding. B16-F10 cells were plated on plates and maintained. Then medium in each well was substituted by fresh medium. Materials (HPI, PEI-FPBA, PEI 1.8 K and PEI 25 K) coated with labeled HSP70-shRNA plasmids were added and cultured for another 1 h. Immediately, cells were harvested and suspended with fresh PBS for next flow cytometry (FCM) (AECE, U.S.A.) analysis (n = 3). For the competitive assay, B16-F10 cells were advanced to incubate with excess HA to bind to CD44 receptors overexpressed on the cell surface followed by adding the complexes[43]. SA receptors were performed following the similar methods.

### Endosomal escape assay

Endosomal escape capability of MGCN was visualized by confocal laser scanning microscope (CLSM) (ZEISS, Germany). B16-F10 cells were plated on glass bottom dishes for 12 h. MGCN containing 2 µg of HSP70-shRNA plasmids labeled with YOYO-1 were incubated with cells. At various time points (0.5 h, 2 h, 4 and 8 h), lysosomes and endosomes were stained with LysoTracker Red and cell nuclei were counterstained with DAPI. Finally, cells were fixed for further examination.

The intracellular distribution of ICG was also determined by CLSM. B16-F10 cells were seed into glass bottom dishes. MGCN was added and incubated with cells, then cell nuclei were stained with DAPI as the manufacturer’s protocol and further examined by CLSM.

### Gene transfection in vitro

B16-F10 cells were seeded to plates overnight. Enhanced green fluorescent protein (EGFP), a reporter gene, was utilized to appraise transfection efficiency. PEI-FPBA/pEGFP or HPI/PEI-FPBA/pEGFP nanocomplexes were incubated with B16-F10 cells for 6 h. PEI 1.8 K, PEI 25 K and Lipofectamine 3000 loading with EGFP plasmids were used as controls according to product’s protocols. Then the culture medium was substituted by fresh medium, cells were further maintained for another 24 h. Transfection experiments were repeated at least three times. Fluorescence images were directly observed by inverted fluorescent microscopy (Olympus, Japan). Transfection efficiency and mean fluorescence intensity (MFI) were analyzed by using FCM.

### Evaluation of gene silencing efficiency of MGCN in B16-F10 cells

HSP70-shRNA containing a hairpin loop was designed at https://www.ncbi.nlm.nih.gov/gene/15525. The sequences of the primers were 5’-GCTCTTGCTTATGGAATCTAT-3’, 5’-CGGGCATAAAGGTTACATATA-3’ and 5’-CACAGAGAATGAGGGTAAGAT-3’. The sequences were synthesized and constructed into the vectors provided by Fubio Biotechnology Co. Ltd. (Jiangsu, China). Then MGCN containing HSP70-shRNA was transfected to B16-F10 cells, transfection method was outlined previously.

Subsequently, RNA was extracted from the cultured cells using the Trizol and then transcribed into cDNA for qPCR. The following mouse primers were used: HSP70, forward primer 5’-TTTCAGAGCTGCTATGTCGCT-3’ and reverse primer 5’-TTGGCATTAGAAATTACCTGGCT-3’; 3-phosphate dehydrogenase (GAPDH), forward primer 5’-AGGTCGGTGTGAACGGATTTG-3’ and reverse primer 5’- GGGGTCGTTGATGGCAACA-3’. TSINKE® Master qPCR Mix (Beijing, China) was utilized for qPCR. Each sample was repeated at least three times. HSP70-shRNA plasmids with the most effective silence effect were selected for follow-up experiments.

### Western blot analysis

B16-F10 cells were transfected with MGCN containing HSP70-shRNA for 24 h, and received or did not receive 808 nm irradiation (2 W/cm^2^, 5 min). Then total proteins from the cultured cells were extracted using RIPA lysis buffer (Invitrogen, U.S.A.) and quantitated through the BCA assay. Protein was separated by SDS-PAGE (10%) and then it was transferred to PVDF membranes. Finally, the PVDF membranes were incubated at 4℃ with primary antibody overnight and conjugated to horseradish peroxidase for chemiluminescence detection.

### Photothermal toxicity of MGCN in vitro

B16-F10 cells were plated on plates for 12 h. The DMEM medium with various concentrations (0 µg/mL − 20 µg/mL) of HPI/PEI-FPBA/ HSP70-shRNA nanocomplexes was added. Fresh medium was replaced after 24 h and B16-F10 cells were received 808 nm irradiation (2 W/cm^2^, 5 min). Cells received HPI/PEI-FPBA/ HSP70-shRNA – Light and HPI/PEI-FPBA/Control-shRNA (abbreviated as Con-shRNA which was used as a control and does not induce interference effect against HSP70) + Light treatments were used as controls. 12 h later, cell viabilities were detected by MTT assay. Photothermal toxicity of MGCN was also evaluated using LIVE/DEAD kit and visualized with inverted fluorescence microscope.

### Apoptosis analysis in vitro

Annexin V-FITC/PI kit was used for identifying the cell apoptosis ratio. B16-F10 cells were divided into follow groups, transfected with different complexes and received different treatments: (1) Blank; (2) HPI/PEI-FPBA/HSP70-shRNA – Light; (3) HPI/PEI-FPBA/Con-shRNA + Light; and (4) HPI/PEI-FPBA/HSP70-shRNA + Light. Cells in group (3) and group (4) were given irradiation (2 W/cm^2^, 5 min). Then cells were continued to culture for another 12 h and collected for apoptosis assay using FCM. Each experiment was performed in triplicate.

### Photothermal response of MGCN in vitro and in vivo

MGCN containing different concentrations of ICG was dispersed in aqueous solution and received irradiation, free ICG and PBS were used as controls. Infrared thermographic images and real time temperature were documented using infrared thermal imaging instrument (Magnity, China).

C57BL/6 mice were used to establish subcutaneous melanoma model. B16-F10 cells were injected in the right back flanks. Mice were randomized to three groups when tumors volume reached 300–400 mm^3^. Then, 100 µL of NS, HPI/PEI-FPBA/Con-shRNA and HPI/PEI-FPBA/HSP70-shRNA were injected into mice *via* the tail vein. After injection, tumors were exposed to laser irradiation. Meanwhile, infrared thermographic images and real time temperature were documented as described previously.

### Biodistribution and tumor accumulation of MGCN

To investigate the tropism and tumor accumulation ability of HPI/PEI-FPBA/HSP70-shRNA nanocomplexes in vivo, A375 cells were used to establish subcutaneous melanoma xenograft models for melanin of B16-F10 cells and hair of C57BL/6 mice having influence on imaging [44]. Briefly, A375 cells were injected to the right back flanks. Nanocomplexes were injected into the mice intravenously when tumors volume reached 300–400 mm^3^. Then fluorescence images were conducted at 2 h, 4 h, 8 and 24 h. Tumors and major organs were obtained for fluorescence imaging ex vivo at 24 h post the injection.

### Antitumor evaluation

The B16-F10 subcutaneous melanoma model was constructed firstly. Mice were randomized into five groups receiving different treatments (n = 5): (1) NS; (2) HPI; (3) HPI/PEI-FPBA/HSP70-shRNA - Light; (4) HPI/PEI-FPBA/Con-shRNA + Light; and (5) HPI/PEI-FPBA/HSP70-shRNA + Light. Mice in group (4) and group (5) were exposed to laser irradiation. Tumor volume and body weight were monitored. At the end of treatments, mice were sacrificed. Blood was harvested for serum biochemistry. The subcutaneous tumors and major organs were collected for hematoxylin and eosin (H&E) staining, TUNEL assay and immunohistochemistry (IHC) analysis, respectively.

### Statistical analysis

Quantitative results were expressed as mean ± SD. One-way ANOVA analysis and student’s t-test performed statistical analysis. Significant differences were presented as asterisks in figures: *P < 0.05, **P < 0.01 and ***P < 0.001.

## Results and discussions

### Preparation and characterization of MGCN

The core-shell structured MGCN was constructed in a two-step electrostatic interacted way. Firstly, materials of PEI-FPBA were synthesized by the condensation reaction of FPBA and PEI 1.8 K (Fig. S1). The products were analyzed by ^*1*^* H* NMR and FT-IR. Both the proton peaks and peak areas for FPBA, PEI 1.8 K and PEI-FPBA were observed in ^*1*^* H* NMR **(Fig. S2A)**. The characteristic absorption of B-O appeared at 1344 cm^− 1^, absorption of C=O appeared at 1680 cm^− 1^ and absorption of ―NH―CO― appeared at 1570 cm^− 1^ in FT-IR **(Fig. S2B)**. Hence, FPBA successfully conjugated to PEI 1.8 K. The average number of FPBA moieties conjugated to PEI 1.8 K was calculated using ^*1*^* H* NMR analysis. According to the integrals of peak areas, the average grafting number of FPBA was calculated to be 1.6 for PEI-FPBA1, 2.6 for PEI-FPBA2, 3.4 for PEI-FPBA3 and 4.9 for PEI-FPBA4, respectively (Table S1). Subsequently, the transfection efficiency of different grafting number of PEI-FPBA was examined on B16-F10 cells. As the grafting number of FPBA increased, the transfection efficiency first increased then decreased, the most efficient polymer PEI-FPBA2 could transfect nearly 90% in B16-F10 cells compared with PEI 25 K and PEI 1.8 K **(Fig. S3)**. Due to the excellent performance, PEI-FPBA2 was chosen to form PEI-FPBA/pDNA (mass ratio of 10:1, defined as core) in the subsequent experiments. Secondly, the shell of MGCN was consisted of HA with further modification of PEG and ICG (HA-PEG-ICG, HPI). Synthetic route of HPI was depicted in **Fig. S4**. The ^*1*^* H* NMR and FT-IR results indicated that HPI was successfully synthesized **(Fig. S5)**.

**Fig. 1:**
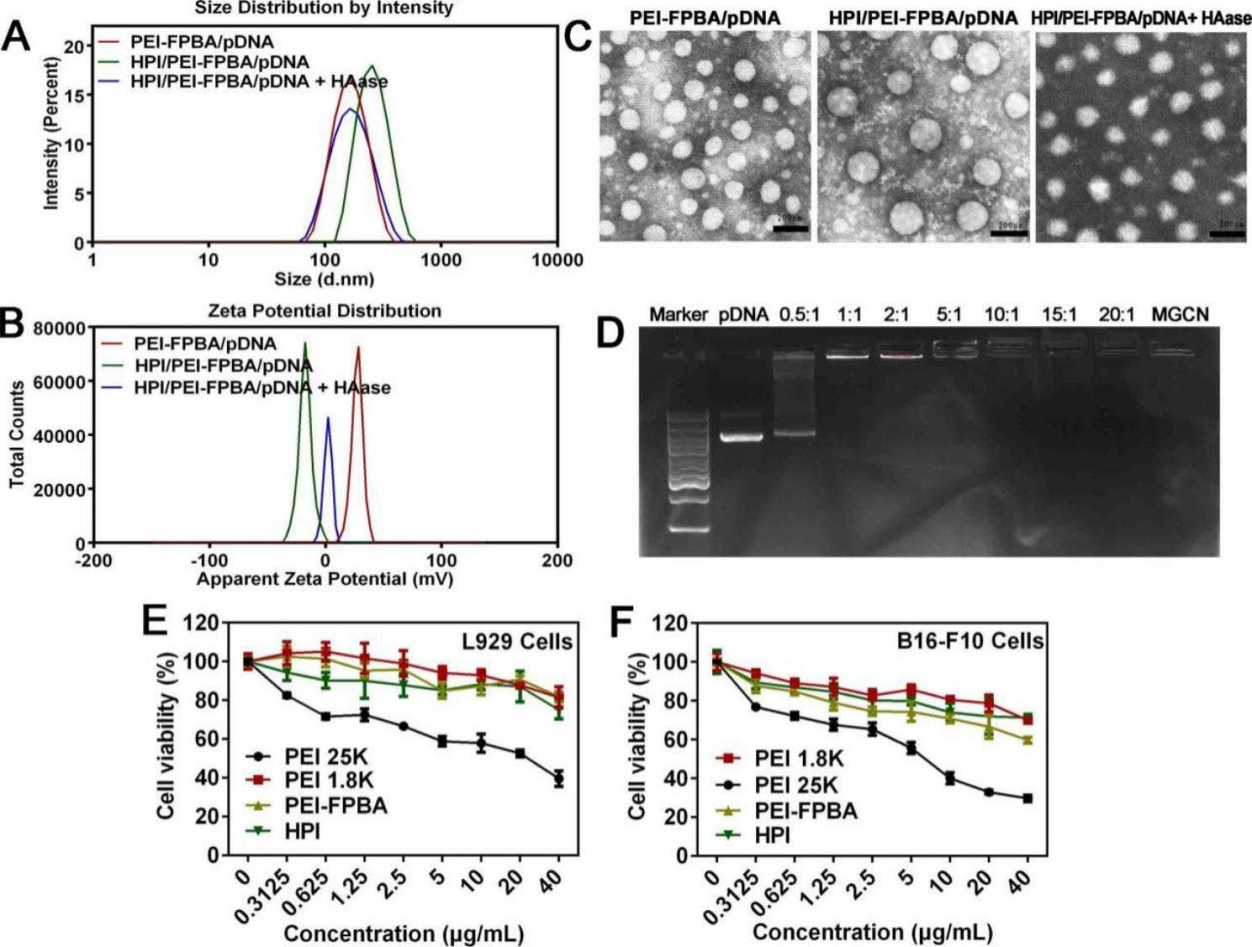
Preparation and Characterization of MGCN. **A.** Hydrodynamic diameter and (**B**) zeta potential of PEI-FPBA/HSP70-shRNA, HPI/PEI-FPBA/HSP70-shRNA and HPI/PEI-FPBA/HSP70-shRNA + HAase nanocomplexes. **C.** Morphologies of PEI-FPBA/HSP70-shRNA, HPI/PEI-FPBA/HSP70-shRNA and HPI/PEI-FPBA/HSP70-shRNA + HAase nanocomplexes measured by TEM. The scale bar represented 200 nm. **D.** Agarose gel retardation assays of the core and MGCN at different mass ratios. Lane 1, DNA ladder; Lane 2, naked HSP70-shRNA plasmids; Lane 3–9, PEI-FPBA/HSP70-shRNA with mass ratios of 0.5:1, 1:1, 2:1, 5:1, 10:1, 15:1, 20:1; Lane 10, HPI/PEI-FPBA/HSP70-shRNA. Cell viability after treated with PEI 1.8 K, PEI 25 K, PEI-FPBA and HPI in (**E**) L929 cells and (**F**) B16-F10 cells

To prepare core-shell structured MGCN, we first constructed PEI-FPBA/HSP70-shRNA complexes as the core *via* mixing HSP70-shRNA plasmids with PEI–FPBA at a mass ratio of 1:10 through electrostatic interaction, then followed by the addition of shell to form MGCN at a final mass ratio of 1:40 between HSP70-shRNA plasmids and HPI. The core showed average hydrodynamic diameter of 163.4 ± 2.0 nm and positive zeta potential of 29.2 ± 2.7 mV. After the core was coated with shell, the complexes had average hydrodynamic size of 271.3 ± 4.1 nm and negative zeta potential of -17.8 ± 0.6 mV (Fig. 1A, B). Such reversal was attributed to the successful coating of HPI at the particle surface. In addition, both core and MGCN had spherical morphologies under TEM (Fig. 1C). In order to verify the responsiveness of MGCN for hyaluronidase (HAase), MGCN was treated with HAase. Results clearly showed that average hydrodynamic size of MGCN had decreased to 187.2 ± 9.0 nm and zeta potential reversed to 4.5 ± 0.4 mV (Fig. 1A, B). Morphologies of MGCN were changed as demonstrated by TEM observation (Fig. 1C). All results above evidenced that MGCN had agile responsiveness of the enzyme in TME. Subsequently, DNA condensation capacities of PEI-FPBA were evaluated by agarose gel retardation experiment. PEI-FPBA prevented plasmids migration at the mass ratio of 1:1 or above. This result demonstrated that PEI-FPBA could ensure plasmids loading and coat well with plasmids at a low mass ratio. Moreover, addition of HPI did not affect plasmids condensation with PEI-FPBA (Fig. 1D). MTT assays showed that PEI-FPBA and HPI had lower toxicity than PEI 1.8 K and PEI 25 K, and grafting of FPBA moieties did not enhance the cytotoxicity of PEI-FPBA significantly compared with PEI 1.8 K. Results indicated the materials with no significant toxicity could be used for further gene delivery (Fig. 1E, F).

### Photothermal response ability of MGCN in vitro

**Fig. 2:**
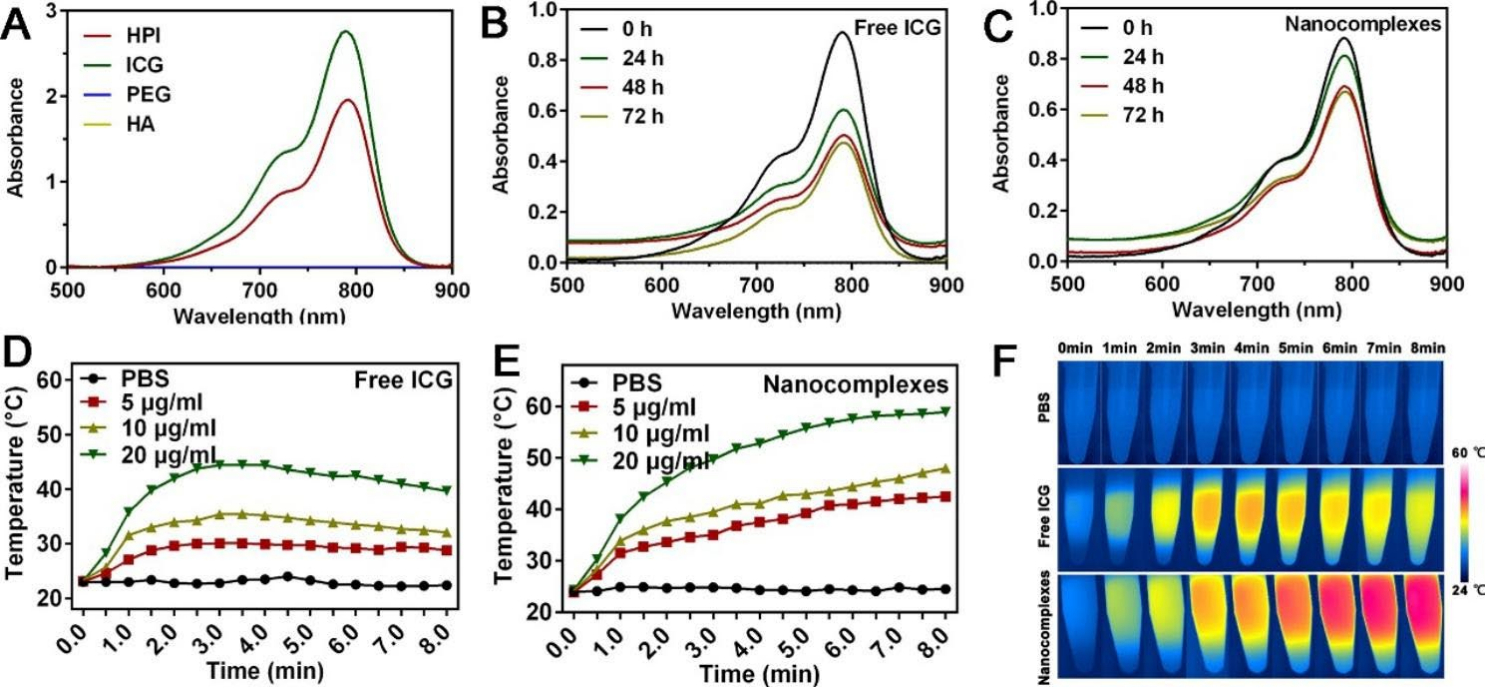
Photothermal response abilities of MGCN. **A.** UV–vis absorption spectra of HPI, free ICG, PEG and HA. Absorption of (**B**) free ICG and (**C**) nanocomplexes. ICG and nanocomplexes were stored for 72 h at 25℃ in the dark, and measured at 0 h, 24 h, 48 h, 72 h in the dark. Temperature rise profiles of (**D**) free ICG and (**E**) nanocomplexes at various concentrations. **F.** Infrared thermographic maps of PBS, free ICG and nanocomplexes (containing 20 µg/mL ICG) recorded by infrared thermal imaging instrument during 808 nm laser irradiation

Photothermal effect of MGCN was the premise for PTT, here, we investigated the photothermal response abilities of MGCN in vitro. Firstly, the absorbance values of HPI and free ICG were detected using UV–vis spectrophotometer. As shown in Fig. 2A, HPI performed well absorption at 780 nm, confirming that ICG had successfully grafted on HPI and its absorption characteristic was still maintained. ICG total content in HPI was 10%. Secondly, we investigated whether MGCN had a higher stability than free ICG. The absorption spectra of MGCN and ICG were recorded in different time intervals after storing at room temperature in the dark. As a result, the absorption intensity of free ICG decreased by 55% after 72 h (Fig. 2B). However, the absorption intensity of MGCN still remained above 70% of initial absorption (Fig. 2C). Possible cause of this delay may be that ICG grafted on MGCN could tighten to each other preventing degradation *via* intermolecular interaction during the formation process of nanocomplexes. Thirdly, in order to assess the photothermal performance of MGCN, we recorded the temperature elevation profiles and infrared thermographic images of MGCN and free ICG under 808 nm laser irradiation. After laser irradiation, MGCN could maintain certain ranges of temperature and possessed better photothermal performance than free ICG (Fig. 2D-F). The higher photothermal performance may be derived from the better stability of MGCN.

### Cellular uptake analysis and endosomal escape of MGCN

**Fig. 3:**
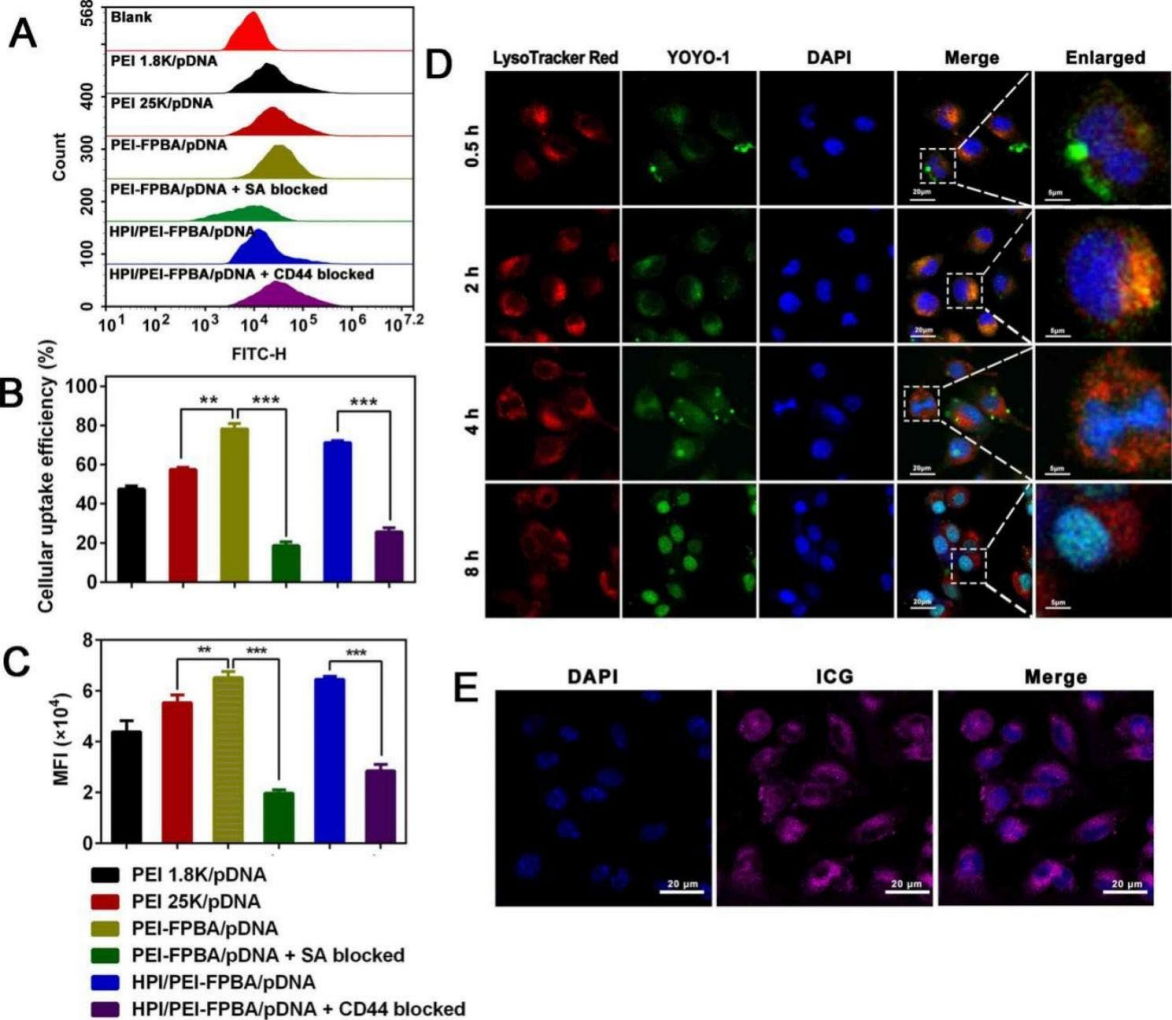
Cellular uptake analysis and endosomal escape of MGCN. **(A)** Cellular uptake analysis of PEI 1.8 K/pDNA, PEI 25 K/pDNA, PEI-FPBA/pDNA, PEI-FPBA/pDNA + SA blocked, HPI/PEI-FPBA/pDNA, and HPI/PEI-FPBA/pDNA + CD44 blocked using flow cytometry in B16-F10 cells. **(B)** Quantitative analysis of cellular uptake efficiency and (**C**) mean fluorescence intensity (MFI) in B16-F10 cells. **D.** The endo/lysosomal escape process of MGCN was shown in B16-F10 cells. Pictures were obtained by CLMS at 0.5 h, 2 h, 4 and 8 h, respectively. HSP70-shRNA plasmids were labeled with YOYO-1 (green), nuclei were counterstained with DAPI (blue), and endo/lysosomes were counterstained with LysoTracker Red (red). The scale bar represented 20 μm. **E.** Cellular uptake and distribution of ICG in B16-F10 cells. The scale bar represented 20 μm. **P < 0.01, ***P < 0.001

For a powerful gene therapy, it is a momentous step for complexes to internalize into cells. PEI-FPBA coated with YOYO-1 labeled plasmids was added to cells for FCM analysis. PEI 1.8 K/pDNA and PEI 25 K/pDNA were used as controls. Results showed that PEI-FPBA/pDNA performed the highest cellular uptake efficiency and strongest MFI intensity among all groups, approximately 77% of PEI-FPBA/pDNA were internalized by cells (Fig. 3A-C). We speculated that FPBA moieties improved complexes affinity to the cell membrane leading to higher cellular uptake efficiency. Previous studies showed that B16-F10 cells had high expression levels of CD44 receptors[46]. HA derivatives were able to react selectively with CD44 receptors and FPBA was able to form reversible covalent cyclic boronic esters with SA receptors, achieving specific targeting function for tumor cells. Based on this, competition assay was carried out to assess whether the cellular uptake of MGCN was certainly associated with CD44 receptors or SA receptors. B16-F10 cells were pre-incubated with excess HA to saturate CD44 receptors and pre-incubated with excess FPBA to saturate SA receptors, respectively. Cellular uptake efficiency of MGCN reduced to 33% and 20% when B16-F10 cells were advanced to culture with excess HA or FPBA, evidencing that MGCN could target tumor cells through ligand-receptor interactions (Fig. 3A-C).

Effective endosomal escape was known as another crucial step for gene therapy, so we investigated the intracellular localization of HSP70-shRNA using CLMS. Since the core of MGCN was made from PEI-FPBA, it was supposed to have effective endosomal escape capability and could release plasmids to the cytosol due to proton sponge effect. As evidenced in Fig. 3D, MGCN attached to the surface of B16-F10 cells at the first 0.5 h and entrapped into lysosomes at 2 h. After incubation for 4 h, MGCN began penetrating into the nuclei, and eventually translocated to the nuclei at 8 h. At the same time, ICG could be observed in cytosol based on self-fluorescence (Fig. 3E). The results clearly indicated that MGCN performed excellent properties of endo/lysosomal escape, allowing efficient expression of HSP70-shRNA and tumor accumulation of ICG for PTT.

### Evaluation of transfection efficiency in B16-F10 cells

**Fig. 4:**
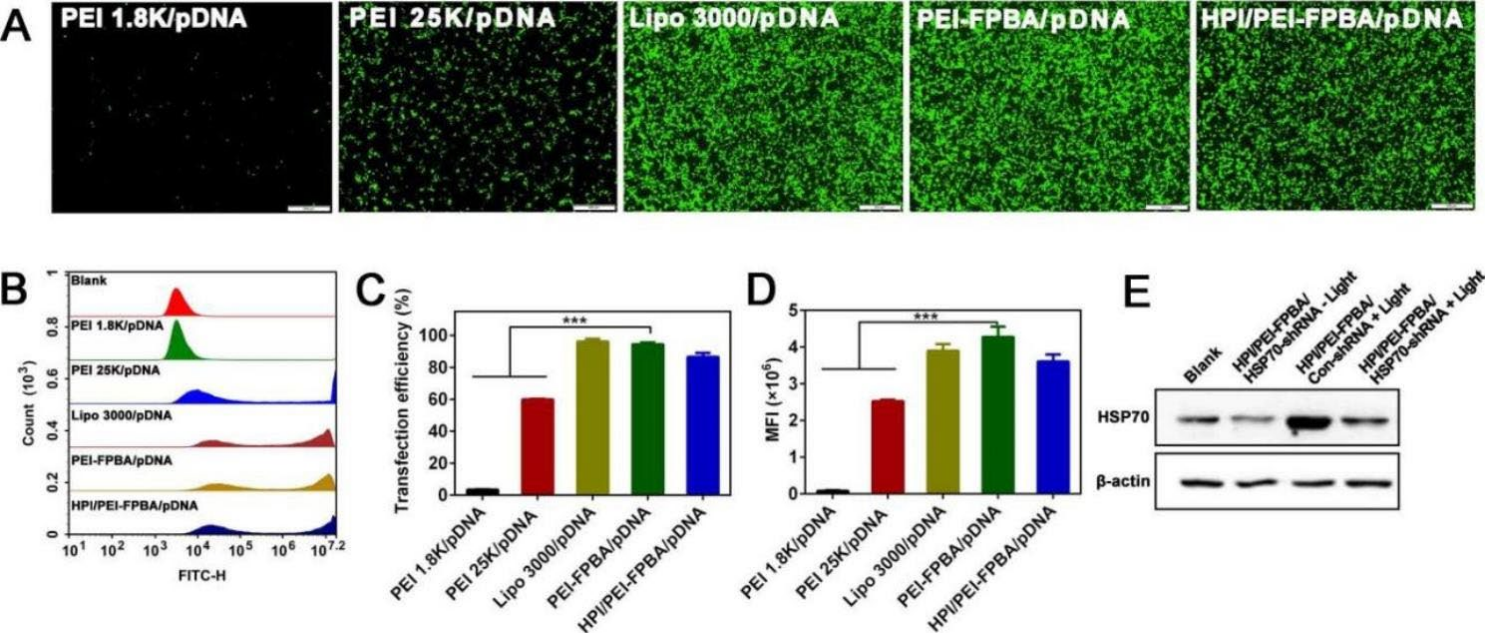
Evaluation of transfection and western blot analysis. **(A)** Fluorescence images of different nanocomplexes taking by fluorescence microscopy at 24 h in B16-F10 cells. The scale bar represented 500 μm. **(B)** The transfection efficiency of nanocomplexes in B16-F10 cells using flow cytometry. **(C)** Transfection efficiency and (**D**) MFI quantification assay in B16-F10 cells. **E.** Expression levels of HSP70 protein. ***P < 0.001

We discussed the transfection efficiency of MGCN in B16-F10 cells. PEI 1.8 K, PEI 25 K and Lipofectamine 3000 loading with EGFP plasmids served as controls (Fig. 4A-D). PEI-FPBA/pDNA performed stronger MFI intensity and higher transfection efficiency (94%) compared with PEI 1.8 K/pDNA (3%) and PEI 25 K/pDNA (60%), and could be comparable to Lipofectamine 3000 (96%) which was regarded as an efficiency commercial transfection reagent. Such excellent transfection efficiency was attributed to the increased cellular uptake and excellent properties of endo/lysosomal escape. At the same time, MGCN also showed transfection capability, which may be related to the specific recognition for CD44 receptors.

### HSP70-shRNA interference efficiency in vitro

Overexpression of HSP70 generated an additional protective effect against heat stress. To this end, we selected HSP70 as a target gene and designed three shRNA sequences for cancer treatment. Targeted sequencing revealed the successful construction of HSP70-shRNA **(Fig. S6A)**. QPCR results indicated that the average interference efficiencies of shRNA1, shRNA2 and shRNA3 were 60%, 55% and 50%, respectively **(Fig. S6B)**. HSP70-shRNA1 with the most effective interference ability was selected for follow-up experiments. Subsequently, MGCN containing HSP70-shRNA1 was transfected to B16-F10 cells, and interference efficiency was confirmed by western blot analysis. As shown in Fig. 4E, levels of HSP70 decreased significantly in HPI/PEI-FPBA/HSP70-shRNA – Light group when cells were transfected with MGCN, which indicated that MGCN disrupted the protein expression efficiently. In addition, levels of HSP70 increased in HPI/PEI-FPBA/Con-shRNA + Light group after irradiation which indeed induced obvious heat shock response. However, levels of HSP70 gained an effective downregulation in HPI/PEI-FPBA/HSP70-shRNA + Light group when cells were transfected with MGCN and then received irradiation. These results collectively demonstrated that PTT indeed induced heat shock response to upregulate the expression level of HSP70 and MGCN could effectively downregulate the expression level of HSP70 *via* HSP70-shRNA.

### Photothermal effect of MGCN in vitro

**Fig. 5:**
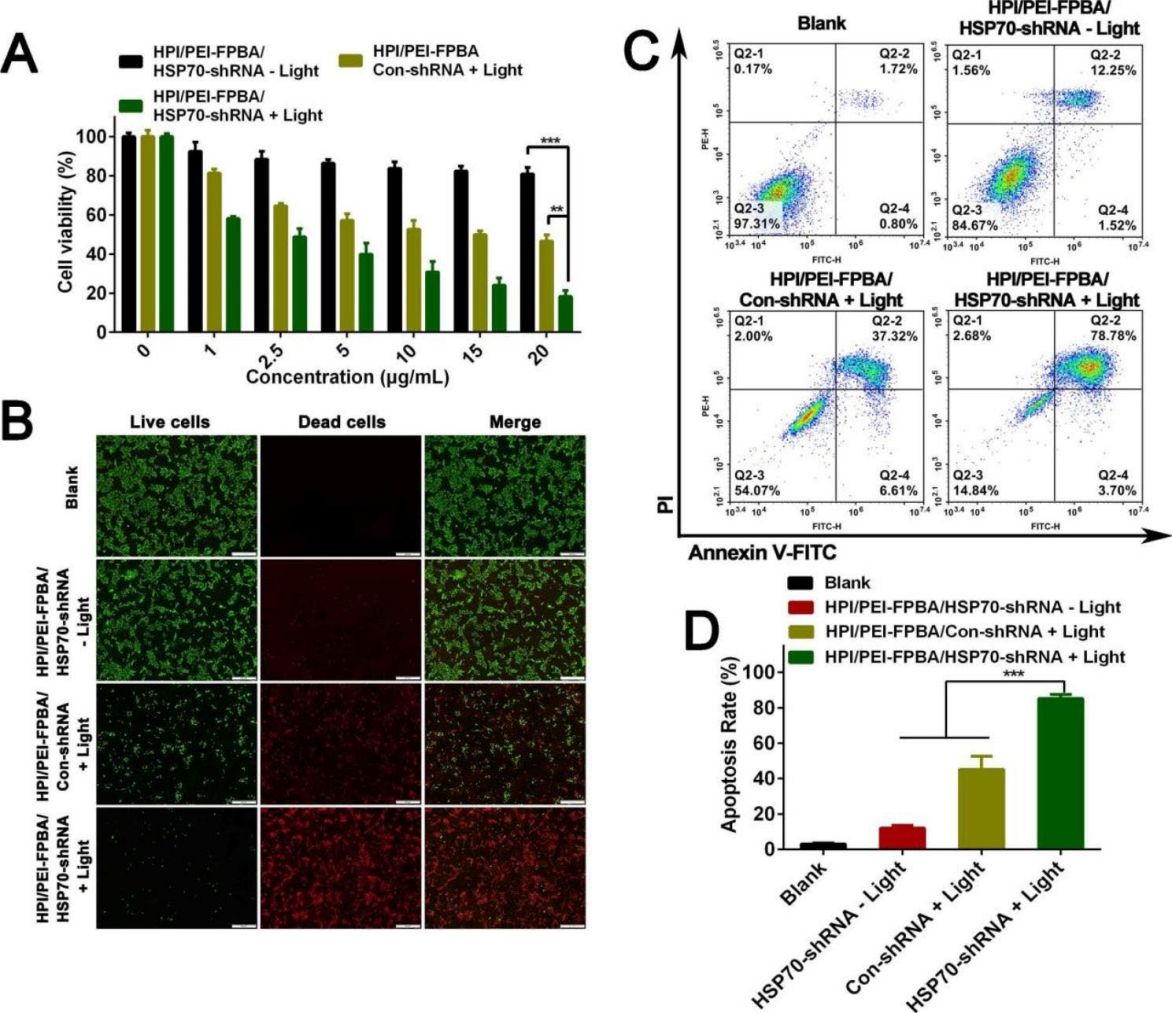
Photothermal effect of MGCN in vitro. After nanocomplex administration, B16-F10 cells were further maintained for another 24 h and then received photothermal therapy. **(A)** Photothermal effect in B16-F10 cells after different treatments measured by MTT. **(B)** Fluorescence images of live/dead staining of B16-F10 cells taken by fluorescence microscopy after treated with different nanocomplexes. Living cells were counterstained with FITC (green), nuclei were counterstained with PI (red). The scale bar represented 500 μm. **(C)** Apoptosis analysis of B16-F10 cells determined with Annexin V-FITC/PI kit by flow cytometry. **(D)** Quantitative analysis of the apoptosis rate in B16-F10 cells. ***P < 0.001

Photothermal effect of MGCN was assessed by MTT assay. In Fig. 5A, cell viability in HPI/PEI-FPBA/HSP70-shRNA – Light group slightly decreased to 85%, cell viability in HPI/PEI-FPBA/Con-shRNA + Light group reduced to 46% after irradiation. More importantly, the cell viability in HPI/PEI-FPBA/HSP70-shRNA + Light group remarkably decreased to 18% after irradiation. These results indicated that MGCN coupled with laser irradiation at 808 nm could produce a higher cytotoxicity and HSP70-shRNA did play a role to silence expression of HSP70. Analogous results were obtained by co-staining of live and dead cells analysis under an inverted fluorescence microscope intuitively (Fig. 5B). Clearly, HPI/PEI-FPBA/HSP70-shRNA – Light group induced cell death slightly and HPI/PEI-FPBA/Con-shRNA + Light group increased more cell death under laser radiation. Additional cell death occurred in HPI/PEI-FPBA/HSP70-shRNA + Light group significantly. Subsequently, we evaluated the apoptosis-inducing effect of MGCN combined with PTT. From the results (Fig. 5C, D), apoptosis was obviously higher in HPI/PEI-FPBA/HSP70-shRNA + Light group (82.4%) than that in HPI/PEI-FPBA/Con-shRNA + Light group (43.9%) and HPI/PEI-FPBA/HSP70-shRNA – Light group (13.7%). HPI/PEI-FPBA/HSP70-shRNA + Light group exhibited a remarkably combinational effect than other groups, indicating that downregulation of HSP70 protein could overcome the thermoresistance of cells and improve photothermal therapeutic outcomes. Therefore, MGCN showed great promise as a nanoplatform for tumor therapy.

### Tumor targeting ability and photothermal efficiency of MGCN

**Fig. 6:**
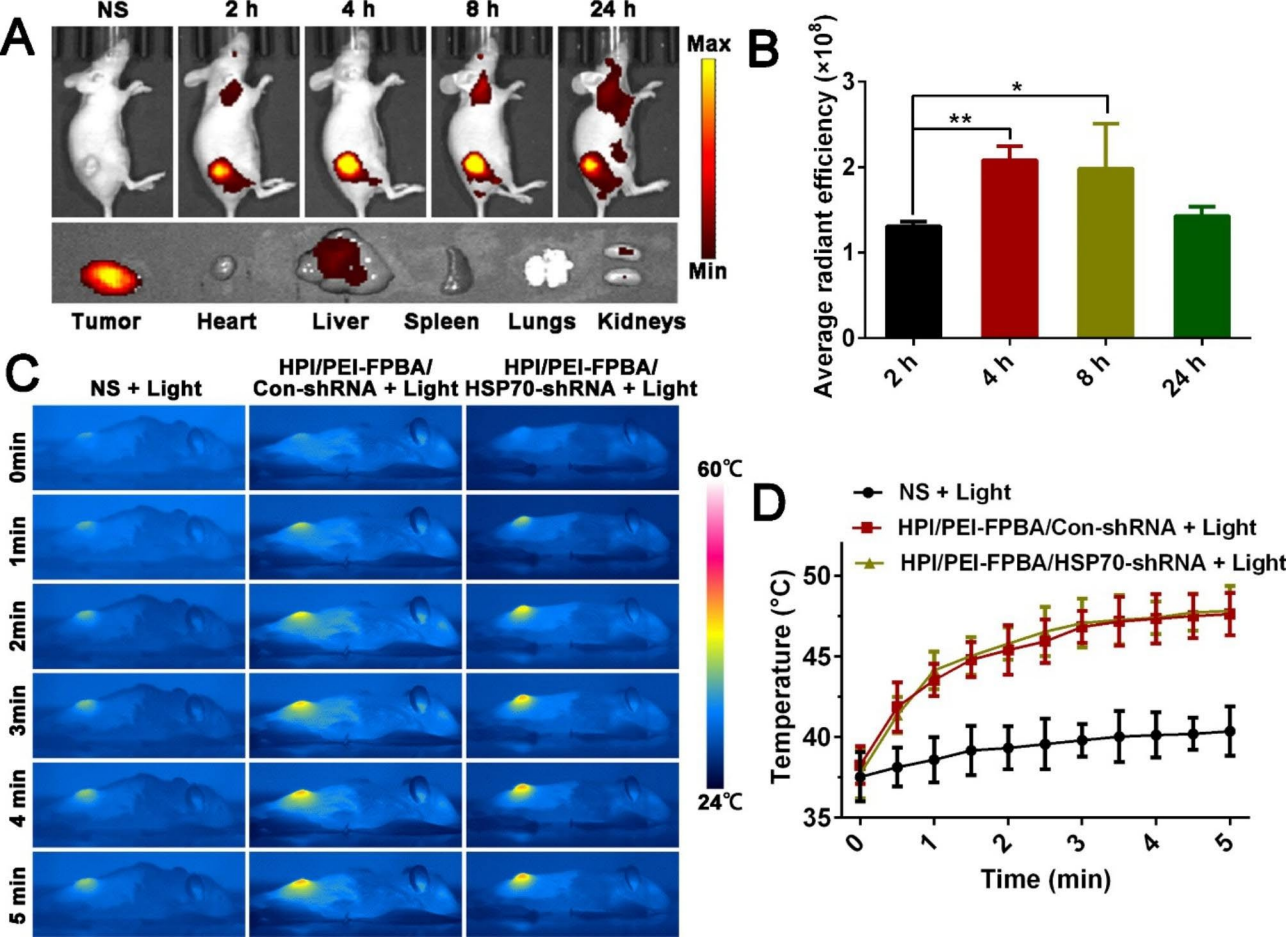
Tumor targeting ability and photothermal efficiency of MGCN in vivo. **A.** Fluorescence images of HPI/PEI-FPBA/HSP70-shRNA nanocomplexes at 2 h, 4 h, 8 h, 24 h and ex vivo fluorescence images of organs harvested from nude mice at 24 h. **B.** Quantitative analysis of the average radiant efficiency, *P < 0. 1, **P < 0.01. **C.** Infrared thermographic maps and (**D**) Temperature elevation curves in tumor-bearing mice treated with irradiation for 5 min. Mice were injected with saline, HPI/PEI-FPBA/Con-shRNA and HPI/PEI-FPBA/HSP70-shRNA *via* the tail vein, respectively. After 6 h of injection, tumors were exposed to laser irradiation (808 nm, 2 W/cm^2^, 5 min)

It was crucial to deliver HSP70-shRNA to tumor tissue. MGCN was specially designed to overcome biological hurdles and achieve accumulation in tumor tissue. In vivo tumor targeting ability of MGCN was determined, and fluorescence intensity images of MGCN were acquired at 2 h, 4 h, 8 and 24 h. In Fig. 6A, B, fluorescence signals appeared inside the tumor tissue and were observed at 2 h after the injection. Stronger signals appeared at the tumor tissue over time and enhanced gradually from 4 to 8 h, thereafter, the intensity of fluorescence declined with time. 24 h after injection, mice were sacrificed. Tumors and main organs were harvested. Ex vivo fluorescent images further confirmed accumulation of MGCN in tumor tissue. No appreciable fluorescence signal was presented in heart, spleen, lungs and kidneys. These results demonstrated that MGCN could accumulate at the tumor tissue for specific targeting capability.

To assess the photothermal efficiency of MGCN in vivo, MGCN was injected into mice *via* the tail vein, NS + Light and HPI/PEI-FPBA/Con-shRNA + Light were used as controls. Infrared thermographic images of tumor and real time temperature were documented (Fig. 6C, D). After irradiation, the maximum temperatures of the tumors reached around 47℃ in both HPI/PEI-FPBA/Con-shRNA + Light group and HPI/PEI-FPBA/ HSP70-shRNA + Light group, which can lead to tumor damage.

### Antitumor effect and toxic evaluation of MGCN

**Fig. 7:**
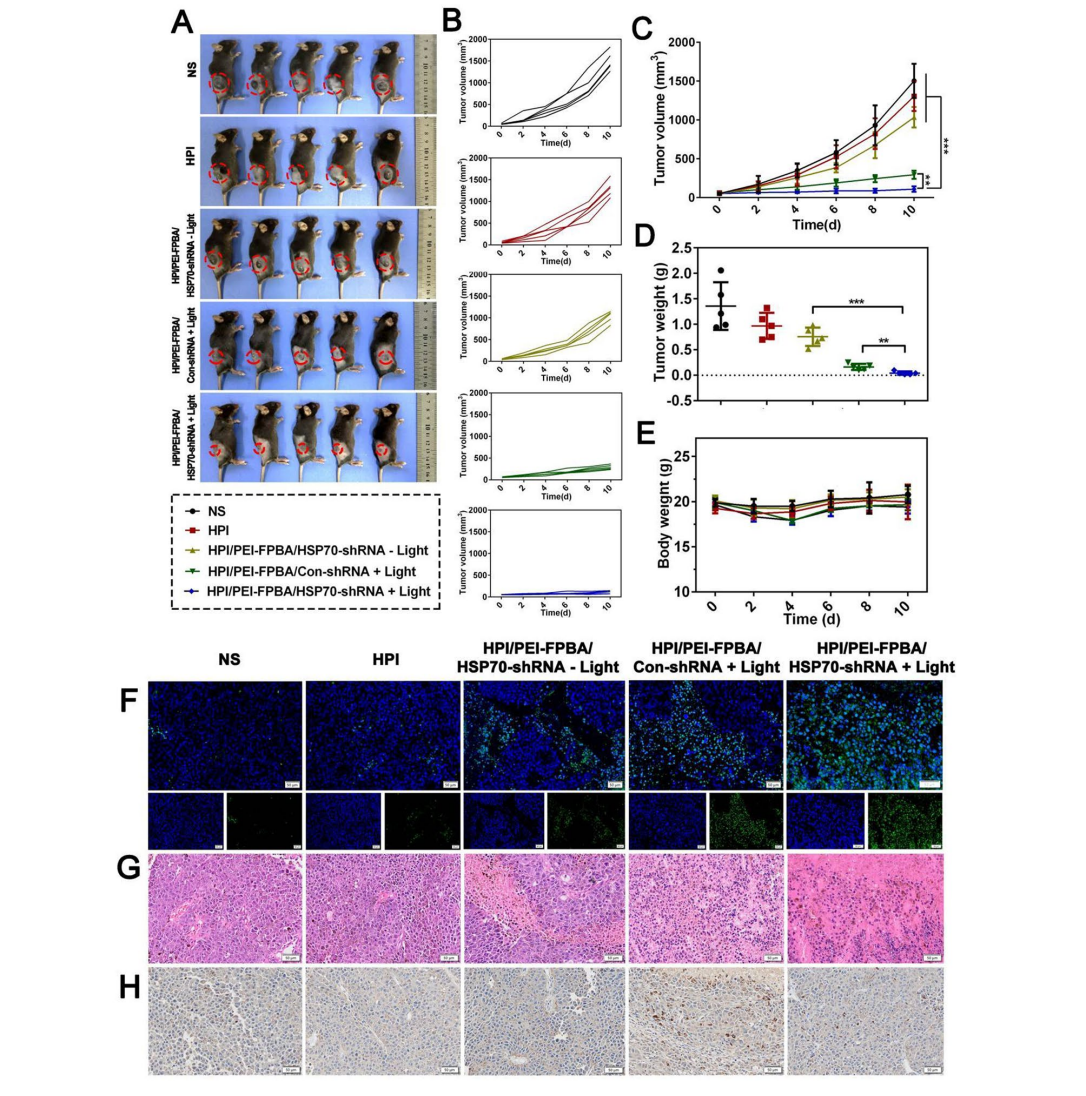
Antitumor effect of MGCN in vivo. **A.** Appearance of tumor after therapy. B16-F10 subcutaneous model was first established and C57BL/6 mice were randomized into five groups with different treatments (n = 5): (1) NS; (2) HPI; (3) HPI/PEI-FPBA/HSP70-shRNA - Light; (4) HPI/PEI-FPBA/Con-shRNA + Light; and (5) HPI/PEI-FPBA/HSP70-shRNA + Light. **B.** Tumor growth curves of mice. Tumor volumes were monitored and individual tumor volumes were plotted. **C.** Tumor volume-time curves. **P < 0.01, ***P < 0.001. **D.** Average tumor weight of tumor-bearing mice. After different treatments, the subcutaneous tumors were removed and weighed. **E.** Body weight change curves of mice. Weight was determined every 2 days. **F.** TUNEL staining in tissue sections from the tumor. Positive cells were stained by FITC (green) and nuclei were stained by DAPI (blue). The scale bar represented 50 μm. **G.** Histological examination of H&E staining of tumor sections. The scale bar represented 50 μm. **H.** Immunohistochemical localization of HSP70 protein. The scale bar represented 50 μm. The positive expression color was tan

Encouraged by outstanding anticancer performance in vitro, we further evaluated the in vivo antitumor effect of MGCN against subcutaneous melanoma. The tumor-bearing mice were treated with 808 nm laser irradiation after intravenously injected (Fig. 7A-E). Overview of final animal experiments, the group of HPI did not show noticeable inhibition of the tumor growth, suggesting that our material had no significant toxicity. Over the treatment period, mice in HPI/PEI-FPBA/HSP70-shRNA - Light group showed a slight inhibition against tumors while mice in HPI/PEI-FPBA/Con-shRNA + Light group showed limited growth inhibition of tumors. However, the tumor growth of mice in HPI/PEI-FPBA/HSP70-shRNA + Light group was strikingly suppressed compared with the control group. These excellent treatment results were benefited from downregulation of HSP70 protein which breaking the thermoresistance of cells. TUNEL assay and H&E staining were utilized to further confirm the antitumor effect in vivo. Histological examination of H&E staining indicated that MGCN combined with PTT effectively inhibited the growth of tumor compared with control groups (Fig. 7G). TUNEL assays were performed to determine apoptotic cells in tumor tissues. More green fluorescent signals were observed from the HPI/PEI-FPBA/Con-shRNA + Light group than the NS group, HPI group and HPI/PEI-FPBA/HSP70-shRNA - Light group, indicating that PTT could induce cell death by activating apoptosis pathways. Furthermore, the numbers of positive cells increased obviously in HPI/PEI-FPBA/HSP70-shRNA + Light group with the same irradiation, indicating that a large number of cells underwent apoptosis (Fig. 7F). It could be concluded that suppression of HSP70 expression could lead into more cell apoptosis. IHC results (Fig. 7H) suggested that levels of HSP70 increased significantly after irradiation in HPI/PEI-FPBA/Con-shRNA + Light group, indicating that PTT indeed induced upregulation of HSP70 in vivo. In contrast, lower expression of HSP70 could be observed in HPI/PEI-FPBA/HSP70-shRNA + Light group, which ascribed to the targeting silencing effect of HSP70-shRNA delivered by MGCN.

After treatment, we investigated the potential toxicity of MGCN in vivo by serum biochemistry and H&E staining. As shown in **Fig. S7**, functional markers remained at normal levels, indicating low toxicity of MGCN. Additionally, histological examination of the H&E staining of vital organs indicated no obverse histopathologic changes compared with control group **(Fig. S8)**. MGCN performed low toxicity in vivo.

## Conclusions

In summary, we had constructed a nanoplatform through a two-step assembly process for breaking the protective mechanism of cellular heat stress response in PTT. On the one hand, MGCN silenced the HSP70 expression induced by PTT, and tumor cells were more susceptible to PTT. On the other hand, MGCN enhanced ICG accumulation in tumor consequently improving photothermal therapeutic outcomes. We demonstrated that MGCN could prolong circulation time in vivo, carry HSP70-shRNA and ICG to tumor regions specifically, response to tumor microenvironment achieving size and charge double-variable transformation, and escape from endo/lysosomal compartments resulting in efficient gene release. Our results showed an excellent antitumor effect of MGCN owning to the cascade augmented synergistic effects of photothermal/gene therapy. This proposed a powerful strategy to overcome thermoresistance and enhance treatment efficacy of PTT.

## Electronic supplementary material

Below is the link to the electronic supplementary material.

Supplementary Material 1

## Data Availability

All data needed to support the conclusions are present in the paper and/or the Supporting Information. Additional data related to this study are available from the corresponding authors upon reasonable.
