# Supplementary material for: A multifunctional ‘golden cicada’ nanoplatform breaks the thermoresistance barrier to launch cascade augmented synergistic effects of photothermal/gene therapy
Source: J Nanobiotechnology. 2023 Jul 17;21:228. doi: 10.1186/s12951-023-01983-3 (PMC10353101; doi:10.1186/s12951-023-01983-3)
Supplement: Supplementary file 1 — Supplementary Material 1 [file 12951_2023_1983_MOESM1_ESM.pdf]

Supporting Information

A Multifunctional 'Golden Cicada' Nanoplatform Break the Thermoresistance Barrier to Launch Cascade Augmented Synergistic Effects of Photothermal/Gene Therapy

Wen Yang [a,1], Ning Wang [a,1], Jin Yang [a], Chao Liu [a], Shuang Ma [a], Xiye Wang [a], Wenzhen Li [a], Meiling Shen [a], Qinjie Wu [a], Changyang Gong [a]**

[a] *Department of Biotherapy, Cancer Center and State Key Laboratory of Biotherapy, West China Hospital, Sichuan University, Chengdu, 610041, P. R. China.*

[1] *These authors contributed equally.*

* To whom correspondence should be addressed (C Gong and Q Wu). E-mail: chygong14@163.com, cellwqj@163.com.

Table S1 Characterization of the PEI-FPBA

Polymer	Average Number [1]
PEI-FPBA1	1.6
PEI-FPBA2	2.6
PEI-FPBA3	3.4
PEI-FPBA4	4.9

[1] According to the integrals of peak areas, the average grafting numbers of FPBA moieties conjugated to PEI 1.8K were calculated using ^{1}H NMR analysis.

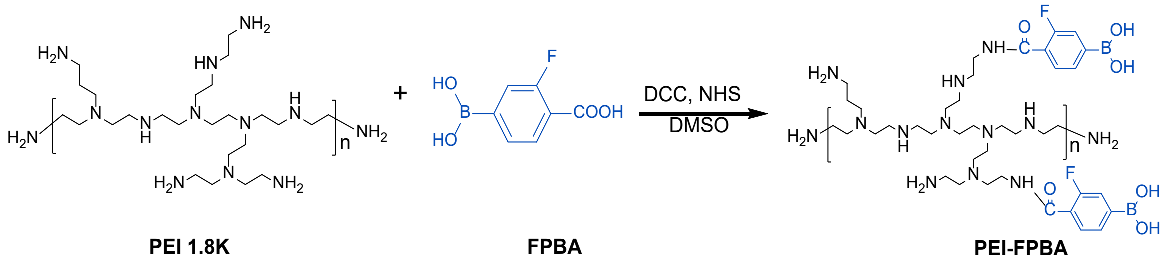

Fig. S1 Synthesis route of PEI-FPBA. PEI-FPBA was synthesized by the condensation reaction of FPBA and PEI 1.8K as denoted in the legend.

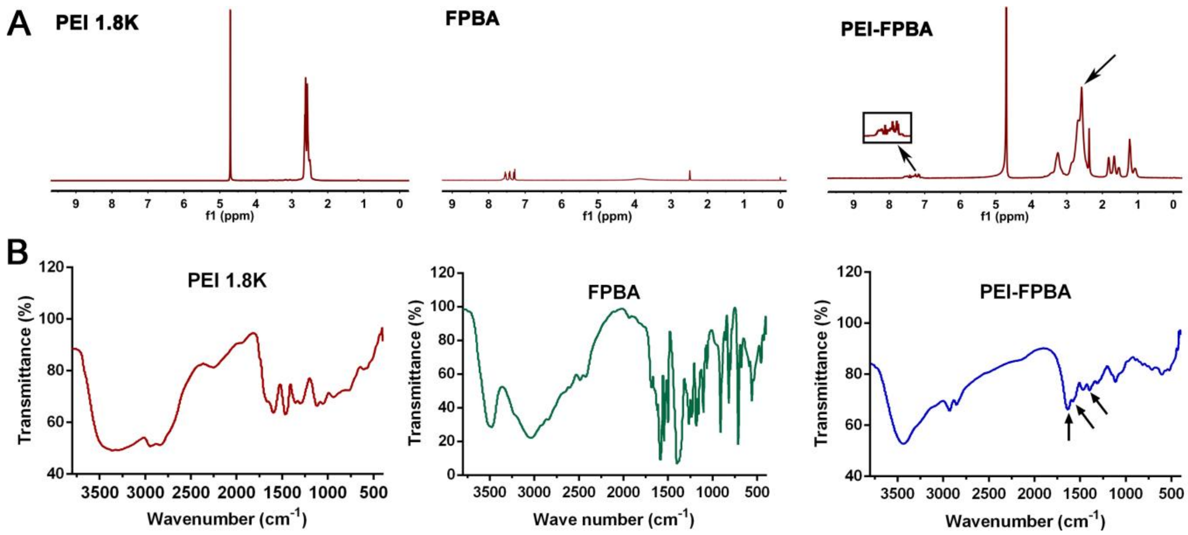

Fig. S2 Characterization of PEI-FPBA. ^{1}H NMR spectrum of (**A**) PEI 1.8K, FPBA, and PEI-FPBA. Chemical shifts 7.0-7.8 ppm for phenyl proton signals and 2.0-3.0 ppm for ethylene proton signals. FT-IR spectrum of (**B**) PEI 1.8K, FPBA, and PEI-FPBA. The characteristic absorption of B − O appeared at 1344 cm^{-1}, absorption of C = O appeared at 1680 cm^{-1}, absorption of —NH—CO— appeared at 1570 cm^{-1}.

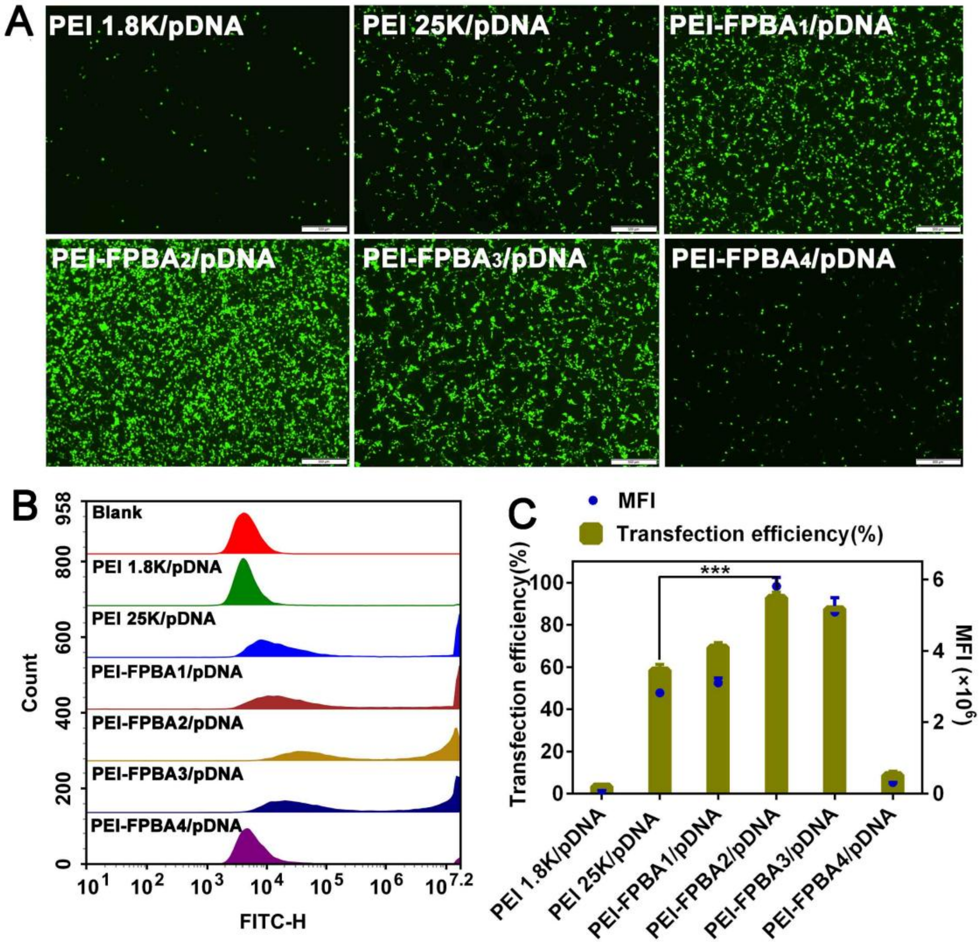

Fig. S3 Transfection efficiency of PEI-FPBA/pDNA in B16-F10 cells after 24h. **A** The fluorescence images of different nanocomplexes taken by fluorescence microscopy at 24h in B16-F10 cells. The scale bar represented 500 μm. **B** The transfection efficiency of nanocomplexes in B16-F10 cells determined by flow cytometry. **C** Transfection efficiency and MFI quantification assay in B16-F10 cells. ***$P < 0.001$.

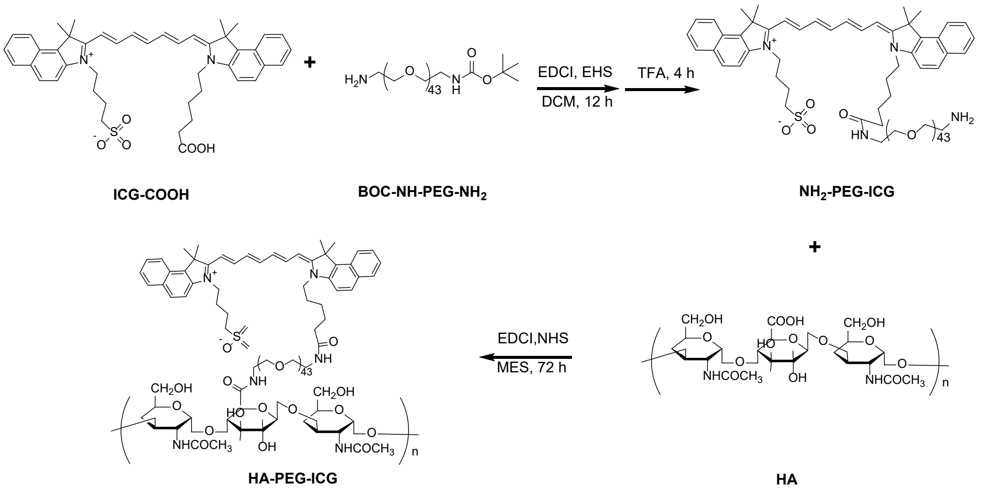

Fig. S4 Synthesis route of HA-PEG-ICG (HPI).

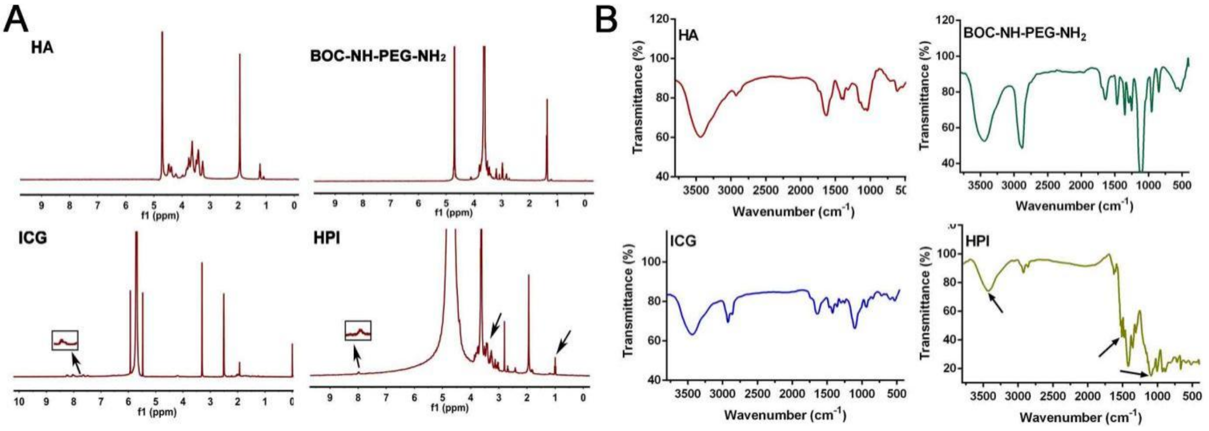

Fig. S5 Characterization of HA-PEG-ICG. ^{1}H NMR spectrum of (**A**) HA, BOC-NH-PEG-NH_2, ICG and HPI. Chemical shifts 7.7-8.2 ppm for ICG proton signals, 1.0-1.3 ppm for HA proton signals, and 3.0-4.0 ppm for PEG proton signals. FT-IR spectrum of (**B**) HA, BOC-NH-PEG-NH_2, ICG and HPI. The characteristic absorption of C = O appeared at 1680 cm^{-1}, absorption of −COOH appeared at 3500 cm^{-1}, absorption of —O— appeared at 1150 cm^{-1}, absorption of phenyl ring appeared at 1650 cm^{-1}.

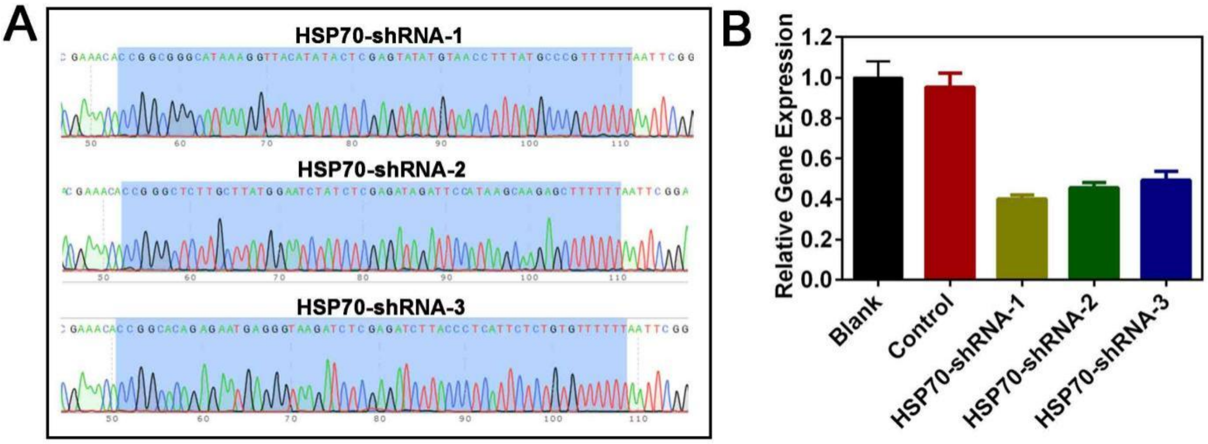

Fig. S6 **A** Construction of HSP70-shRNA plasmids and sequencing results of HSP70-shRNA-1, HSP70-shRNA-2 and HSP70-shRNA-3. **B** Relative gene expression determined by qPCR after B16-F10 cells were transfected with different HSP70-shRNA plasmids.

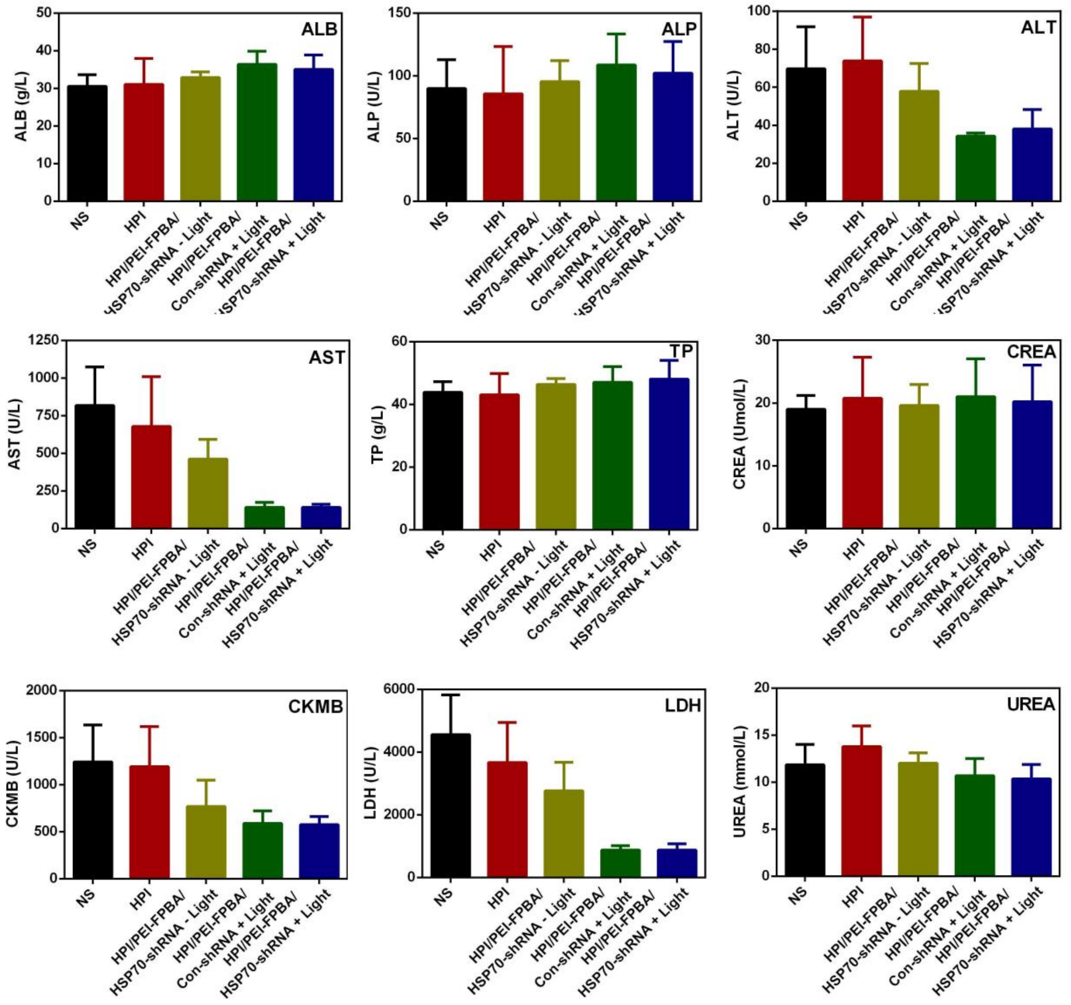

Fig. S7 Biochemical analysis of serum in each group. Mice were sacrificed at the end of treatment, and blood were collected form each group as follow: (1) NS; (2) HPI; (3) HPI/PEI-FPBA/HSP70-shRNA - Light; (4) HPI/PEI-FPBA/Con-shRNA + Light; (5) HPI/PEI-FPBA/HSP70-shRNA + Light.

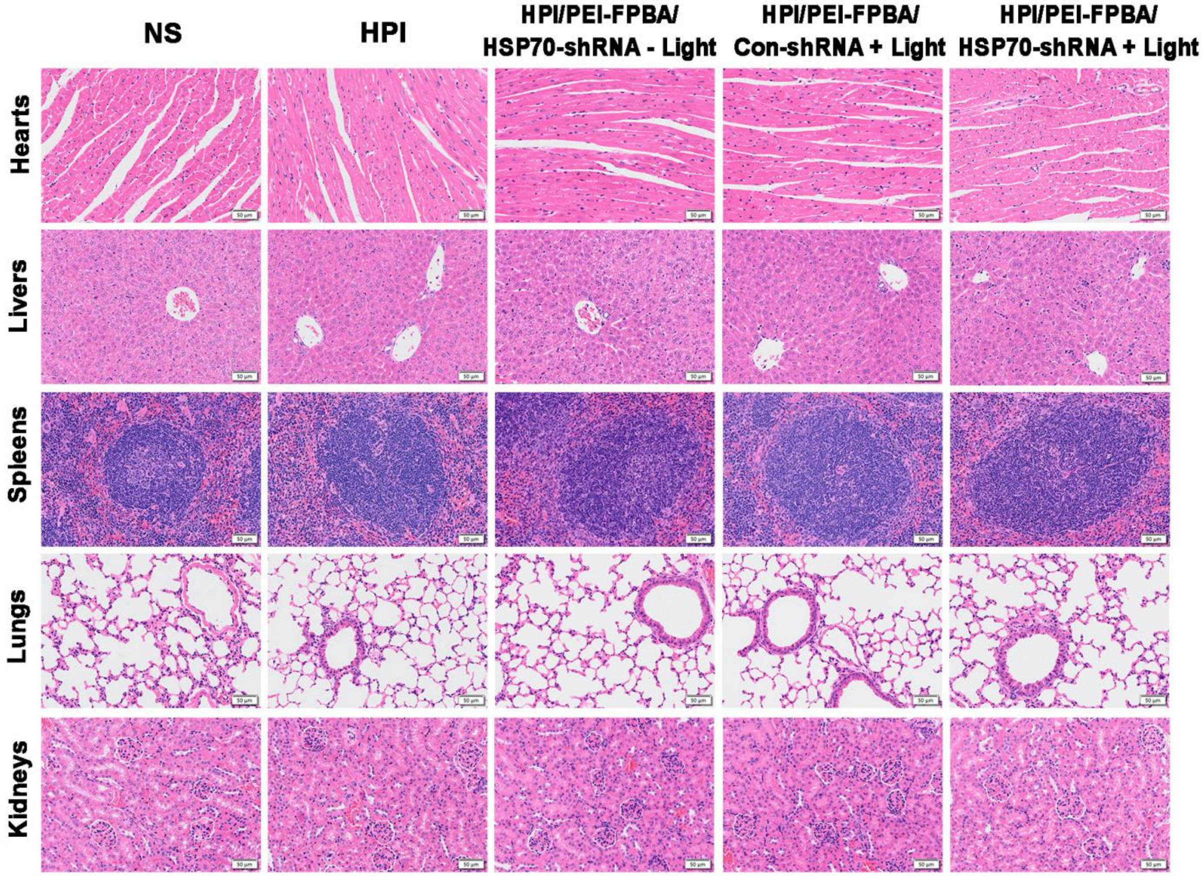

Fig. S8 Histological examination of H&E staining of vital organs of mice taken from groups as follow: (1) NS; (2) HPI; (3) HPI/PEI-FPBA/HSP70-shRNA - Light; (4) HPI/PEI-FPBA/Con-shRNA + Light; (5) HPI/PEI-FPBA/HSP70-shRNA + Light. The scale bar represented 50 μm.
